# Reliability and validity of the Chinese mandarin version of PedsQL™ 3.0 transplant module

**DOI:** 10.1186/s12955-016-0545-0

**Published:** 2016-10-05

**Authors:** Ying Chang, Yanhui Luo, Yuchen Zhou, Ruixin Wang, Na Song, Guanghua Zhu, Bin Wang, Maoquan Qin, Jun Yang, Yuan Sun, Chunfu Li, Xuan Zhou

**Affiliations:** 1 Hematology Oncology Center, Beijing Key Laboratory of Pediatric Hematology Oncology, National Key Discipline of Pediatrics, Ministry of Education; Hematology Oncology Center, Beijing Children’s Hospital, Capital Medical University, 56 Nanlishi Road, Beijing, 100045 China; 2 Beijing Daopei Hospital, Beijing, 100049 China; 3 The First Affiliated Hospital of Southern Medical University, Guangzhou, 510000 Guangdong China

**Keywords:** Reliability, Validity, Chinese version, PedsQL™ 3.0 transplant module

## Abstract

**Background:**

Long-term health-related quality of life (HRQoL) of pediatric patients after hematopoietic stem cell transplantation (HSCT) is increasingly studied worldwide. However, few studies have been performed in China, where no uniform scale is available; the PedsQL™ Cancer Module 3.0 Chinese Mandarin version has been used to evaluate HRQoL of patients after HSCT in China. This study aimed to assess the reliability and validity of the Chinese Mandarin version of PedsQL™ 3.0 Transplant Module.

**Methods:**

Patients between 2 and 18 years old, who underwent HSCT from January 2006 to June 2014, were recruited in Beijing Children’s Hospital affiliated to Capital Medical University, the First Affiliated Hospital of Southern Medical University and Beijing Daopei Hospital. 207 parent reports and 182 child self-reports of the PedsQL™ 3.0 Transplant Module Chinese Mandarin version were assigned, of which 362 were returned.

**Results:**

No missing item response was observed in the returned reports. Cronbach’s alpha coefficient exceeded 0.7 in total scale and every dimension. The intraclass correlation coefficient exceeded 0.8 in all dimensions of child self-reports and parent reports. Spearman’s rank correlation coefficients of items and their respective dimensions were 0.6-0.94 in parent reports, and 0.62-0.93 in child self-reports, while a weak association was found between the items and other dimensions. Exploratory factor analysis indicated a good extraction effect, and construct validity of the scale was >60 %.

**Conclusions:**

The Chinese Mandarin version of PedsQL™ 3.0 Transplant Module has good feasibility, reliability and validity. Its use may help improve the HRQoL of children after HSCT in China.

## Background

Hematopoietic stem cell transplantation (HSCT) is the main therapy for many hematological diseases. With the development of the HSCT technology and stem cell resources, pediatric patients are increasingly achieving long-term survival [1, 2]. However, various adverse events that can affect the health-related quality of life (HRQoL) of children [3–8] can occur during HSCT, and are increasingly studied worldwide.

Health is defined as not only the absence of disease or weakness, but also good physical, psychological and social adaptation of an individual. The medical model has advanced from the biomedical to a bio-psycho-social medical model. The assessment of quality of life (QoL) can better reflect new concepts of health and the medical model, effects of tumors and chronic diseases, and whole human body health.

The focus of HRQoL research is to develop suitable measurement scaling systems [9]. Pediatric Quality of Life Inventory™ (PedsQL™) was initiated in 1987 by Professor James Varni from San Diego Children’s Hospital, and developed into a mature HRQoL system with ideal reliability and validity by repeated evaluation and application. Previously, its Cancer Module (PedsQL™ Cancer Module 3.0) in Chinese Mandarin [10], was used to evaluate HRQoL of patients after HSCT in China. However, these patients have many unique features, including changes of appearance due to long term anti-graft-versus-host disease (GVHD) medicines, anxiety over the chance of relapse, and pain from GVHD that cannot be accurately reflected by cancer scales. The scale also cannot assess patients who suffer from benign disorders. This suggests that a Chinese Mandarin version of the Pediatric Quality of Life Inventory™ 3.0 Transplant Module (PedsQL™ 3.0 Transplant Module), which can guide doctors in assessing patients’ pain both in terms of physiology and psychology by accurate evaluation of HRQoL, is needed. Interestingly, it was shown that PedsQL™ 3.0 Transplant Module could be understood by both children and their parents, with a good correlation between child self-reported surveys and those obtained in parent proxy reports [10]. In this study, we assessed the reliability and validity of the Chinese Mandarin version of PedsQL™ 3.0 Transplant Module in a larger population.

### Subjects

Patients who received HSCT between January 2006 and June 2014 in Beijing Children’s Hospital affiliated to Capital Medical University, the First Affiliated Hospital of Southern Medical University and Beijing Daopei Hospital, were recruited. All patients with no developmental delay, which may affect their HRQoL, and who met the criteria of > 4 month survival post-transplantation, age between 2–18 years old, and all parents able to communicate with the researchers in Chinese, were evaluated. This study was approved by the Ethics Committee of the above three hospitals, and all participants provided signed informed consent.

The patients’ clinical data were collected initially, and trained investigators subsequently performed the survey. Some surveys were completed by email or phone, indicating that the investigators were not completely blinded to patient’s contact information, although there were unaware of gender, age and disease type. All investigators signed an agreement to protect patient’s privacy, and all profiles were kept by the principal investigator.

## Methods

### Measures

#### The Chinese mandarin version of PedsQL™ 3.0 transplant module

We received authorization for using the Chinese Mandarin version of PedsQL™ 3.0 Transplant Module after signing an agreement with its original author, Professor James Varni from Mapi Research Trust. This scale includes child self-reports and proxy reports [8]. The child self- report forms are specific for ages 5–7, 8–12, and 13–18 years, respectively. Parent proxy-report forms are specific for children of ages 2–4, 5–7, 8–12, and 13–18 years, respectively.

The Chinese Mandarin version of PedsQL™ 3.0 Transplant Module [8] has eight dimensions and 46 items, which include About Medicine I (nine items), About Medicine II (eight items), Transplantation and Others (eight items), Pain and Hurt (three items), Worry (seven items), Treatment Anxiety (four items), How I look (three items) and Communication Problems (three items). Each item assesses the frequency of occurrence in the past month. A 5-point response scale was utilized across child self-report forms for ages 8–18 and parent proxy-report forms: 0 (100 points) = never a problem; 1 (75 points) = almost never a problem; 2 (50 points) = sometimes a problem; 3 (25 points) = often a problem; 4 (0 points) = almost always a problem. To further ease the use of young child self-report forms (ages 5–7), the response scale was reworded and simplified to a 3-point scale: 0 (100 points) = not at all a problem; 2 (50 points) = sometimes a problem; 4 (0 points) = a huge problem. Facial expressions (happy, normal, and sad) were also used in this scale to help with understanding. In one dimension, the sum of all item scores divided by the number of items was considered the score for that dimension. The sum of total item scores divided by the number of total items was considered to be the score of the whole scale. The higher the score, the better the HRQoL.

#### Procedure

The physician introduced the questionnaire and its purpose to the patients and their parents. Investigators were trained to help the children and their parents complete the questionnaire. Some patients had returned to their hometowns, and the questionnaires were completed by email or by phone. In this case the investigators asked the questions to child and one parent separately by phone, allowing self-reports and proxy reports to be assessed separately. The questionnaire was completed only by email if the patients were aged between 2–4 years and did not provide self-reports.

#### Statistical analysis

SPSS 19.0 was used for data processing and statistical analysis. Completion rate and the percentage of missing items were used to evaluate the feasibility of the scale, which was considered invalid if there were > 50 % missing items [8, 9, 11–20]. Cronbach’s alpha coefficient was used to examine the scale’s internal consistency [8, 9, 11–20]. Intraclass correlation coefficient between child self-reports and parent proxy reports was used to assess test-retest reliability. Spearman’s rank correlation coefficients of each item in its dimension and the scale were used to determine content validity, while construct validity was evaluated by exploratory factor analysis.

## Results

### General information

A total of 207 children met the criteria for inclusion in the study. 207 parents were assessed by parent proxy reports, and 182 children completed the self- report forms; 25 were excluded from self-report because they were aged between 2 and 4 years. However, some patients had returned to their hometowns, and the questionnaires had to be completed by email or phone. Some parents were reluctant for their children to answer the questionnaires over the phone. Finally, 155 children completed the questionnaires (Table 1).

**Table 1:** Age and gender distribution of reports

	Parent proxy report (*n* = 207)	Child self-report (*n* = 155)
Age		
2–4 years	25 (12.1 %)	0
5–7 years	74 (35.7 %)	56 (36.1 %)
8–12 years	72 (34.7 %)	68 (43.9 %)
13–18 years	36 (17.4 %)	31 (20 %)
Gender		
Male	124 (69.4 %)	87 (56.1 %)
Female	83 (30.6 %)	68 (43.9 %)

### Feasibility

In this study, 207 parent proxy report forms of PedsQL™ 3.0 Transplant Module Chinese Mandarin version were sent out, all of which were returned. A total of 182 child self-report forms were sent out, of which 155 were returned. No missing item response was found in any returned report.

### Reliability

Cronbach’s alpha coefficient was used to assess the scale’s internal consistency, with ≥ 0.7 representing good reliability. Tables 2 and 3 show that Cronbach’s alpha coefficients were > 0.7 in all dimensions, as well as the total scale in parent proxy reports and child self-reports.

**Table 2:** Cronbach’s α coefficients of parent proxy reports in each dimension and the total scale

Field	Items	Cases	Cronbach’s α coefficient
	(No.)	(No.)	
Scale	46	207	0.942
About Medicine I	9	207	0.916
About Medicine II	8	207	0.817
Transplantation and Others	8	207	0.795
Pain and Hurt	3	207	0.778
Worry	7	207	0.918
Treatment Anxiety	4	207	0.903
How I Look	3	207	0.803
Communication Problems	4	207	0.93

**Table 3:** Cronbach’s α coefficients of child self-reports in each dimension and the total scale

Field	5–7 years age group			8–18 years age group			Scale		
	Items	Cases	A	Items	Cases	α	Items	Cases	α
	(No.)	(No.)		(No.)	(No.)		(No.)	(No.)	
Scale	46	54	0.955	46	101	0.947	46	155	0.954
About Medicine I	9	54	0.929	9	101	0.922	9	155	0.926
About Medicine II	8	54	0.786	8	101	0.825	8	155	0.821
Transplantation and Others	8	54	0.824	8	101	0.855	8	155	0.86
Pain and Hurt	3	54	0.775	3	101	0.739	3	155	0.759
Worry	7	54	0.941	7	101	0.919	7	155	0.929
Treatment Anxiety	4	54	0.979	4	101	0.907	4	155	0.935
How I Look	3	54	0.893	3	101	0.8	3	155	0.834
Communication Problems	4	54	0.963	4	101	0.904	4	155	0.931

*Note*: Child self- reports forms are specific for 5–7, 8–12, and 13–18 year age groups. Data from the 8–12 and 13–18 year age groups was analyzed together because the forms for these two groups are the same

### Content validity

Content validity was evaluated by Spearman’s rank correlation analysis of each item in its dimension and the scale. In this study, the Spearman’s rank correlation coefficients of items and their respective dimensions were 0.6-0.94 in parent proxy reports, and 0.62-0.93 in child self-reports, which indicates a moderate to high association. The association was weak between items and other dimensions.

### Construct validity

Exploratory factor analysis was utilized to examine the construct validity of the scale in this study. Kaiser-Meyer-Olkin (KMO) value was >0.7, achieved by factor analysis of each item, indicating that the scale was suitable for factor analysis. Factor extraction was analyzed by principal component analysis. Ten common factors were extracted, which conformed to the theory structure of the scale and involved every item. Six dimensions were completely extracted in parent proxy reports. The dimensions “About Medicine II” and “Transplantation and Others” were divided into two common factors. The cumulative contribution rate was nearly 70 %, which indicated a good extraction effect. In child self-reports, four dimensions were completely extracted. The dimensions “About Medicine I”, “About Medicine II”, “Transplantation and Others”, and “Pain and Hurt” were divided into six common factors. The cumulative contribution rate was close to 74 %, which showed a good extraction effect.

## Discussion

In this study, we analyzed the reliability and validity of the Chinese Mandarin version of PedsQL™ 3.0 Transplant Module. The results of this large scale analysis showed that the return rates of parent proxy- and child-self reports were 100 and 85.2 %, respectively. The smaller the missing item rate, the better feasibility of the survey; previous studies have found an average missing rate for the PedsQLTM 3.0 Transplant Module of less than 1 % [1, 2]. No missing item response was found in this study, which confirmed that the Chinese Mandarin version of PedsQLTM 3.0 Transplant Module had good feasibility.

Similar to most previous studies, we found good reliability in this study. Cronbach’s alpha coefficients exceeded 0.7 in the total scale and every dimension in both parent proxy reports and child self-reports, indicating good reliability. However, these values were higher than those described in previous reports assessing patients with a wide range of diseases in other countries, with values generally over 0.7 but around 0.6 for some dimensions [13, 16, 18, 21].

Content validity and construct validity were also analyzed in the current study. Content validity determines whether items can represent the content/theme or not. Spearman’s rank correlation coefficient >0.5 indicates a moderate association, while a value > 0.8 suggests a high association. In contrast, values < 0.5 and 0.3 represent weak and very weak associations, respectively. Our results showed moderate to high association between items and their respective dimensions, but weak associations with other dimensions, indicating that the Chinese version has good content validity. Exploratory factor analysis was utilized to examine the construct validity of the scale. Ten common factors were extracted, which conformed to the theory structure of the scale and involved every item. The results showed cumulative contribution rates >60 % in both parent proxy- and child self-reports, indicating a good extraction effect. Thus, the Chinese Mandarin version of PedsQL™ 3.0 Taken together these results indicated that the Transplant Module has good construct validity, in agreement with previous studies. Indeed, PedsQL was originally designed to evaluate health-related quality of life in 2–18 year patients, with the PedsQL 3.0 Cancer Module specific for pediatric cancer [20]. This pioneer work demonstrated that all parent proxy-report scales of PedsQL 3.0 Cancer Module met or exceeded the minimum reliability standard of 0.70, with a proven validity for the module [20]. In addition, the PedsQL 3.0 Transplant Module was used to assess pediatric solid organ transplant recipients of liver, kidney, heart and small bowel, supporting its excellent feasibility, reliability and construct validity in pediatric patients with solid organ transplants [8]. Nevertheless, the PedsQL 3.0 Transplant module was not primarily designed for HSCT. Nevertheless, a previous study showed that both the PedsQL 3.0 solid transplant and HSCT modules were highly correlated [22]. Nevertheless, it is the only PedsQL questionnaire that is currently validated in Mandarin. It is very critical to assess patient experience during treatment for pediatric cancer; several questionnaires, such as PedsQL 4.0, PedsQL 3.0, PedsQL Multidimensional Fatigue Scale, and the Pediatric Inventory for Parents have been used to this end in other countries [22]. Therefore, HSCT specific PedsQL modules, such as PedsQL 4.0 Generic Core Scales, should be evaluated for the assessment of HRQoL in pediatric patients after HSCT in China.

This study has some limitations. The patient information in all cases could not be kept completely anonymous as some patients were surveyed by phone or email; this may have introduced some bias. This study included patients from three hospitals, but a better accuracy of results would be expected from multiple centers throughout China. Collecting patients from only 3 hospitals is likely to have some level of selection bias. When grouping the patients by age, some groups had a small sample size, with a limited number of disease types. More patients from multiple centers would address these concerns, and should be assessed in the future before the Chinese Mandarin version of PedsQL™ 3.0 Transplant Module is applied throughout China.

## Conclusions

The Chinese Mandarin version of PedsQL™ 3.0 Transplant Module showed good feasibility, reliability and validity. This specific scale may provide more accurate data to analyze the impact of various factors on HRQoL than previous assessments, and may help improve the HRQoL of children after HSCT in China.

## Abbreviations

HRQoL: Health related quality of life
HSCT: Hematopoietic stem cell transplantation
KMO: Kaiser-Meyer-Olkin
PedsQL™ 3.0 Transplant Module: Pediatric quality of life inventory™ 3.0 transplant module
QoL: Quality of life
